# Risk and protective factors for mental ill-health in elite para- and non-para athletes

**DOI:** 10.3389/fpsyg.2022.939087

**Published:** 2022-09-02

**Authors:** Lisa S. Olive, Simon M. Rice, Caroline Gao, Vita Pilkington, Courtney C. Walton, Matt Butterworth, Lyndel Abbott, Gemma Cross, Matti Clements, Rosemary Purcell

**Affiliations:** ^1^Elite Sports and Mental Health, Orygen, Parkville, VIC, Australia; ^2^Centre for Youth Mental Health, The University of Melbourne, Melbourne, VIC, Australia; ^3^School of Psychology, Faculty of Health, Deakin University, Geelong, VIC, Australia; ^4^Athlete Wellbeing and Engagement, Australian Institute of Sport, Canberra, ACT, Australia; ^5^Paralympics Australia, Sydney, NSW, Australia

**Keywords:** Paralympic, mental health, psychology, elite athlete, psychological safety, eating disorder, alcohol use and alcohol problems, psychological distress

## Abstract

**Objective:**

To apply a socioecological approach to identify risk and protective factors across levels of the “sports-ecosystem,” which are associated with mental health outcomes among athletes in para-sports and non-para sports. A further aim is to determine whether para athletes have unique risks and protective factor profiles compared to non-para athletes.

**Methods:**

A cross-sectional, anonymous online-survey was provided to all categorized (e.g., highest level) athletes aged 16 years and older, registered with the Australian Institute of Sport (AIS). Mental health outcomes included mental health symptoms (GHQ-28), general psychological distress (K-10), risky alcohol consumption (AUDIT-C) and eating disorder risk (BEDA-Q). Risk and protective factors across multiple levels of the socioecological model, including individual, microsystem, exosystem and macrosystem level factors were assessed *via* self-report.

**Results:**

A total of 427 elite athletes (71 para and 356 non-para athletes) participated in the study. No significant differences in the rates of mental health problems were observed between para and non-para athletes. Both differences and similarities in risk and protective factor profiles were found across the multiple levels of the sports-ecosystem. Weak evidence was also found to support the hypothesis that certain risk factors, including experiencing two or more adverse life events in the past year, sports related concussion, high self-stigma, inadequate social support and low psychological safety conferred a greater risk for poorer mental health outcomes for para athletes in particular.

**Conclusion:**

Risk factors occurring across various levels of the sports ecosystem, including individual, interpersonal and organizational level risk factors were found to be associated with a range of poorer mental health outcomes. The association between mental ill-health and certain risk factors, particularly those at the individual and microsystem level, appear to be greater for para athletes. These findings have important implications for policy and mental health service provision in elite sports settings, highlighting the need for more nuanced approaches to subpopulations, and the delivery of mental health interventions across all levels of the sports ecosystem.

## Introduction

There is growing interest in elite athlete mental health, which is reflected in a rapidly developing evidence-base (Rice et al., 2016; Gouttebarge et al., 2019; Reardon et al., 2019; Kuettel and Larsen, 2020; Poucher et al., 2021). A recent systematic review suggested that approximately one third of currently competing athletes report experiencing symptoms of the common mental health disorders (e.g., depression and anxiety; Gouttebarge et al., 2019), a rate that is comparable (or elevated for general distress) to the general population (Purcell et al., 2020). In addition, knowledge of sports-related risk factors for mental ill health in elite athletes are becoming increasingly understood (Rice et al., 2019; Kuettel and Larsen, 2020; Walton et al., 2021). However, the evidence to date has been based on study samples comprised largely of non-para athletes, meaning less is known about the prevalence, characteristics and factors that may impact upon athletes from para sports (herein termed para athlete) mental health.

Of the few studies investigating para athlete mental health, most have been qualitative in design, relying on small sample sizes and have often used non-standardized measures of psychopathology (Macdougall et al., 2016; Swartz et al., 2019), which limits our ability to draw strong conclusions. Earlier work by our group, aiming to address some of these limitations and the lack of comparative data on the mental health of para- and non-para athletes, examined the prevalence and correlates of mental health symptoms among these subgroups (Olive et al., 2021). Findings from this study indicated that mental health and wellbeing symptoms were comparable between the para and non-para athlete subgroups, with the exception of para athletes reporting lower alcohol consumption and lower self-esteem (Olive et al., 2021). These findings further showed that a similar proportion of para and non-para athletes indicated they experienced mental health symptoms at a level that would usually warrant a need for professional healthcare (37 and 33%, respectively). For para athletes, this finding was at odds with findings from individuals with impairments from the general (non-athletic) population, where rates of mental health symptoms are often reported to be higher for people with disabilities (Watson et al., 2014).

Along with the urgent need to broaden studies on athlete mental health to include para athlete populations, there is a further need to extend investigations beyond individual-level risk and protective factors for mental health (Purcell et al., 2019). Predominant conceptualizations in elite sport have tended to take the view that mental ill-health is a problem existing with the individual athlete, often ignoring the wider socioecological factors that may be influential in contributing to, or perpetuating mental ill-health (Rice et al., 2022). This interpretation is problematic as it may lead to pathologizing the individual athlete while ignoring important relationships between individual-level factors (e.g., coping, attitudes, substance use) and the broader social and cultural contexts in which they exist (Bronfenbrenner, 1992). This may be particularly relevant when it comes to para athletes, who are likely to experience a range of additional impairment-specific stressors occurring at these broader organizational, cultural and social levels of the “sports ecosystem” (e.g., discrimination, issues with para-sport classification, appropriate access to training facilities or venues), which have the potential to compromise their mental wellbeing (Bundon and Hurd Clarke, 2014; Arnold et al., 2017). Similarly, para athletes may also be exposed to additional factors that are protective of their mental health that are related to their status as an elite para athlete and the Paralympic movement, which may not be afforded to individuals with impairments from the general population (Macdougall et al., 2016; Powell and Myers, 2017).

Bronfenbrenner’s (Bronfenbrenner, 1977, 1986) socioecological model of health emphasizes multiple interacting layers of influence across the “ecosystem.” This model appears to have utility in elite sport, as outlined in a comprehensive framework for athlete mental health (Purcell et al., 2019, 2022). When applied to the elite sporting context, it is likely that mental health outcomes among elite athletes from both para and non-para sports are related to risk and protective factors at various socio-ecological levels, including individual (e.g., age, gender, coping skills), interpersonal (termed the microsystem; e.g., social support, athlete/coach relationship), individual sport (termed the exosystem; e.g., characteristics of the sport and competition, sporting cultures that prioritize performance over wellbeing), and community/society (termed the macrosystem; e.g., public and social media, national/international sporting context) factors. Understanding risk and protective factors within the broader “ecology” of elite sporting environments can inform novel “systems level” interventions that are less prominent in elite sports setting when it comes to early intervention for mental health problems. This may be particularly relevant with regards to identifying risk and protective factors relating to the culture of sporting organizations.

Sporting organizational culture has become a topic of great interest in the context of elite athlete mental health, with a particular interest in psychological safety. Psychological safety was a term first defined in the context of organizational psychology (Schein and Bennis, 1965; Edmondson, 1999) but has more recently been adapted for elite sporting contexts. A recent systematic review aiming to provide conceptual clarity of the term psychological safety in the context of sport described it as group level construct that is perceived (and reported) at an individual level (Vella et al., 2022). The International Olympic Committee define psychological safety as “environments where athletes feel safe in taking interpersonal risks within the sports ecosystem, feel accepted as an integral part of the sports ecosystem, and feel respected by the sports ecosystem” (International Olympic Committee, 2021). With reference to athlete mental health, this may include having sufficient knowledge of mental health concerns (e.g., mental health literacy) and actively promoting cultures of safety for those experiencing symptoms of mental ill-health, which allow them to engage with appropriate intervention. Due to the increased openness and vulnerability between team members that is characteristic of psychologically safe sporting environments, such cultures may act as a protective factor against mental ill-health at the broader exosystem-level of the “sports ecosystem” (Vevoda et al., 2016; Ma et al., 2021), however, this is yet to be fully tested. Our research group has identified that sports psychological safety domains of a newly developed scale, the Sports Psychological Safety Inventory, were inversely related to general and athlete-specific psychological distress, and positively associated with psychological wellbeing among elite athletes (Rice et al., 2022). Hence, providing preliminary evidence for the potential effectiveness of targeting these broader exosystem-level factors (and specifically, psychological safety) for improving athlete mental health.

The aim of this study is to apply a socioecological approach to identify risk and protective factors across each level of the “sports-ecosystem,” which are associated with mental health outcomes among para and non-para athletes; and further, to determine whether para athletes have unique risks and protective factor profiles compared to their non-para counterparts. It is hypothesized that those experiencing greater levels of risk factors and fewer protective factors will have poorer mental health outcomes. Given that rates of mental health symptoms in the general (e.g., non-athletic) population are reported to be higher amongst people with disabilities than in individuals without a disability, it is further hypothesized that the relationships between risk and protective factors and mental health outcomes will be dependent on para athlete status.

## Materials and methods

### Participants and study design

All elite para- and non-para athletes aged 16 years and over, who were supported by the Australian Institute of Sport (AIS) *via* being contracted with a national sporting organization (NSO) were invited to participate. Athletes supported by the AIS receive numerous benefits, ranging from monetary support in the form of grants through to access to high performance resources (e.g., training facilities, technology, equipment and personnel) and access to mental health and wellbeing services. All eligible athletes were invited to participate, *via* either SMS or email, in an anonymous, online cross-sectional survey considering their mental health and wellbeing. On average, the survey took 15–20 min to complete. The only exclusion criteria for the current study was age and ability to read and understand English. All athletes were provided with information regarding the purpose of the study and the method of providing consent to participate (which was implied by participants choosing to begin the survey), prior to commencing the survey. Participants completed the survey between March and May 2020. The study was approved by The University of Melbourne Human Ethics Research Committee (#13718).

### Measures

#### Demographics and background information

Participants were asked to provide a range of basic demographic information (e.g., age, gender, education, employment, accommodation, relationship status, sexual orientation) and background information relating to their role in elite sport. For example, athletes were asked about their selection status for the 2020 Tokyo Paralympics/Olympics (which took place in 2021 due to the COVID-19 pandemic), as well as their main sporting activity over the last month (e.g., actively engaged, injured/adapted training program, illness, on a break) and number of years as an NSO supported athlete. Para athletes were also asked about the nature of their impairment (e.g., physical, visual, intellectual, other), how long ago they had acquired their impairment, their current classification and if there were issues other than classification that were impacting their mental health.

#### Mental health outcomes

##### Mental health symptoms and probable caseness

Mental health symptoms and probable caseness was assessed using the 28-item General Health Questionnaire (GHQ-28; Goldberg and Hillier, 1979), which provides a total score and four subscale scores (somatic complaints, anxiety and insomnia, social dysfunction and severe depression). Higher scores on the GHQ-28 indicate greater mental health symptoms. The GHQ-28 also yields a threshold for “caseness,” defined as symptoms that adversely affect quality of life and are of a level frequently found among individuals seeking help from health professionals (Goldberg et al., 1997).

##### General psychological distress

The Kessler 10 (K10; Kessler et al., 2002), a 10-item screening tool, was used to assess general psychological distress. Participants are asked to rate the frequency with which they experienced psychological distress (e.g., nervousness, hopelessness, fatigue) over the last 4 weeks on a 5-point scale ranging from 1 “none of the time” to 5 “all of the time.” Higher scores represent greater psychological distress.

##### Risky alcohol consumption

The Alcohol Use Disorder Identification Tool-Condensed Version (AUDIT-C; Bush et al., 1998) was used to assess risky alcohol consumption. The AUDIT-C is a brief, three item measure assessing frequency and quantity of alcohol consumption. Each item is scored 0–4, which yields a total score ranging from 0 to 12. Higher scores indicate more risky alcohol consumption.

##### Eating disorder risk

The Brief Eating Disorder in Athletes Questionnaire (BEDA-Q; Martinsen et al., 2014), comprising 9-items, was used to determine eating disorder risk. The first six items ask participants about eating-disorder symptoms (e.g., “I feel extremely guilty after overeating,” “I am preoccupied with the desire to be thinner,”), and are scored on a scale of 0–3 (3 = always, 2 = usually, 1 = often, 0 = sometimes, 0 = rarely, 0 = never), with item 4 reverse-scored. This yields a total score ranging from 0 to 18. In addition, the BEDA-Q includes 3 items on dieting: “Are you trying to lose weight now”? (“yes,” “no”); “Have you tried to lose weight during your career”? (“yes,” “no”); “If yes, how many times have you tried to lose weight (1–2, 3–5, or > 5 times)”?

#### Socioecological risk and protective factors

##### Individual level factors

*Adverse life events* were assessed for the past 12-months and lifetime. This 13-item measure asks participants to endorse (yes/no) if they have experienced general adverse events (e.g., “A person close to me died”) and sport-specific events (e.g., “I felt under-valued or under-paid”; “I was stalked by a fan”). *Sleep* was assessed using the Athlete Sleep Screening Questionnaire (ASSQ: Samuels et al., 2016), a 5-item measure that asks about satisfaction with recent sleep quality, sleep duration, sleep onset latency, sleep maintenance and use of sleep medication. ASSQ total scores (range = 0–17) can be categorized into levels of sleep disturbance (5–7 = mild disturbance, 8–10 = moderate disturbance, 11–17 = severe disturbance; Bender et al., 2018). Experience of *sports related concussion* was assessed *via* two items, reported concussion (yes/no) and number of concussions where applicable. *Social media use* was assessed in terms of hours per day spent on social media. Participants were also asked whether they were satisfied with their life balance (e.g., managing sport, work, social life, family, sleep, etc.) on a dichotomous scale (yes/no). S*elf-stigma* and *mental health literacy* were assessed *via* the Sports Psychological Safety Inventory (SPSI; S. Rice et al., 2022), an 11-item self-report survey that assesses a broad range of factors related to psychological safety in the elite sporting environment. Participants are asked to rate their agreement with each statement on a 5-point scale (0 = Strongly Disagree, 1 = Disagree, 2 = Neutral/Unsure, 3 = Agree, 4-Strongly Agree). The SPSI yields three subscale scores: Mentally Healthy Environment (4-items), Mental Health Literacy (4-items) and Low Self-Stigma (3 items). Higher total scores indicate greater perceived psychological safety. Participants were also asked “What was your main activity related to your sporting profession in the last month?” to determine if they were actively engaged in their sport (e.g., playing/competing) or not (e.g., current injury/illness, in an adapted training program, on a break from sport or other absences).

##### Microsystem factors (interpersonal factors)

*Social support* was assessed using six questions, which enquired about the presence of adequate social support (yes/no), whether the main source of social support came from within their sport (e.g., coach, teammates) or outside their sport (e.g., friends, family), and their satisfaction with the level of social support they received (response ranging from 1 = totally dissatisfied to 7 = completely satisfied). The remaining three items enquired about social isolation (How often do you feel: (1) that you lack companionship; (2) feel left out; and (3) feel isolated from others). Participants responded on a 3-point scale (1 = hardly ever, 2 = some of the time, 3 = often).

##### Exosystem factors (individual sport factors)

Exosystem factors relating to individual sports included a measure of *psychological safety*, which was assessed using the *Mentally Healthy* subscale of the SPSI as previously described (Rice et al., 2022). Participants answer on a five-point response scale (0 = “Strongly Disagree,” 1 = “Disagree,” 2 = “Neutral/Unsure,” 3 = “Agree,” 4 = “Strongly Agree”) how much they agree with each statement (e.g., *“My sport setting is a safe space to disclose mental health problems”*). Items assessing sport specific characteristics included *sport type* (individual or team sport), *aesthetic sport vs. non-aesthetic sport*, *time spent traveling due to sport*, whether participants had *missed significant personal events due to sport-related travel* (yes/no), and *safety concerns while traveling for sport* (yes/no).

##### Macrosystem factors: (Inter/national sporting environment, media/social media)

Athletes reported on the broader support they received within the “sports ecosystem,” including whether they were supported by a National Institute Network (yes/no; e.g., the AIS, Victorian Institute of Sport), and how long they had been a NSO categorized athlete (number of years). Athletes were also asked whether they had been harassed or abused on social media ever (yes/no) or in the past year (yes/no). Para athletes were also asked about their current classification status (e.g., classified, yet to be classified, recently de-classified, and other).

### Data analysis

Simple descriptive statistics were used to compare participant characteristics, risk factors and outcomes between para and non-para athletes. Crude differences were compared using Pearson chi-squared tests for categorical variables and *t*-tests for numeric variables. To evaluate the association between individual risk factors and outcomes, linear regression models were carried out for each risk factor with each outcome variable, adjusting for age and gender for all participants. To better understand how individual risk factors may potentially impact para and non-para athletes differently, stratified analyses were also conducted in each group separately. The differences between the two groups were further validated using interaction models (including an interaction term between risk factor and para athlete status). Missing data were imputed using 20 imputed datasets and regression results were pooled using Rubin’s rules (Rubin, 1996). Analyses were conducted using R version 4.1.3 (2022-03-10) and missing data imputation was conducted using the mice package V3.14.0. Due to the highly explorative nature of the study, correction for multiple comparisons was not conducted as it increases type II errors for those associations that are not null (Rothman, 1990).

## Results

### Participant characteristics

Four-hundred and twenty-seven (71 para- and 356 non-para athletes) athletes consented to participate in the current study, representing 16.5% of eligible of athletes (20.9% of eligible para athletes) who were registered with the AIS and over 16 years of age at the time the survey was open. A summary of participant demographics and sports-related characteristics are provided in Table 1. The participating athletes were representative of the eligible population in relation to para-status (12.4%) and mean age (23.8 years), however, a higher proportion of women athletes completed the survey (67%). This over representation was largely seen in non-para athletes (71% women vs. 29% men) rather than para athletes (52% women vs. 44% men; 4% not reported). Nine athletes identified as Aboriginal (2%) and none identified as Torres Strait Islander. The majority of athletes identified as heterosexual (89.9%), were actively engaged in their sport at the time of the survey (70.7%), and were either not participating in other forms of work (36.8%) or were participating in paid casual work (33.5%). X% of athletes indicated they had availed themselves of an AIS related mental health and wellbeing service. A number of differences were observed between para and non-para athletes, including that para athletes tended to be older (29.5 years para vs. 22.7 years non-para athletes), and therefore were more likely to have completed tertiary education (50.7 vs. 29%), to have bought and be living in their own home (30.9 vs. 12.7%), and to be married or in a *de facto* relationship (29.6 vs. 16.9%). No significant differences were observed between para and non-para athletes on any of the mental health outcomes (all *p* > 0.05; see Table 2), and probable caseness was similar among both groups (45.1% of para athletes vs. 43.5% of non-para athletes).

**TABLE 1:** Demographic and sports-related characteristics of non-para and para athletes.

	Total (*N* = 427)	Non-para athletes (*N* = 356)	Para athletes (*N* = 71)	*P-value*
**Age (years)**				< 0.001
Mean (*SD*)	23.8 (8.1)	22.7 (6.8)	29.5 (11.4)	
Median (Q1, Q3)	22.0 (18.0, 27.0)	21.0 (18.0, 26.0)	28.0 (20.0, 37.0)	
Missing	6	4	2	
**Gender**				0.007
Men	134 (31.8%)	103 (29.1%)	31 (45.6%)	
Women	288 (68.2%)	251 (70.9%)	37 (54.4%)	
Missing	5	2	3	
**Sexual orientation**				0.424
Heterosexual	384 (89.9%)	322 (90.4%)	62 (87.3%)	
Other	43 (10.1%)	34 (9.6%)	9 (12.7%)	
**Aboriginal and/or Torres Strait Islander (ATSI)**				0.644
No	372 (97.6%)	309 (97.5%)	63 (98.4%)	
Yes	9 (2.4%)	8 (2.5%)	1 (1.6%)	
Missing	46	39	7	
**Country of birth**				0.718
Australia	386 (90.4%)	321 (90.2%)	65 (91.5%)	
Other	41 (9.6%)	35 (9.8%)	6 (8.5%)	
**Relationship status**				0.446
In a relationship	240 (56.2%)	203 (57.0%)	37 (52.1%)	
Not in a relationship	187 (43.8%)	153 (43.0%)	34 (47.9%)	
**Highest level of education**				< 0.001
Tertiary education	139 (32.6%)	103 (29.0%)	36 (50.7%)	
Primary/high school education	287 (67.4%)	252 (71.0%)	35 (49.3%)	
Missing	1	1	0	
**Currently studying**				0.022
No	182 (42.6%)	143 (40.2%)	39 (54.9%)	
Yes	245 (57.4%)	213 (59.8%)	32 (45.1%)	
**Any other work in previous month**				0.435
Not working	157 (36.8%)	128 (36.0%)	29 (40.8%)	
Paid/voluntary work	270 (63.2%)	228 (64.0%)	42 (59.2%)	
**Ever treated for a concussion**	87 (20.4%)	80 (22.5%)	7 (9.9%)	
**Sport type**				0.026
Individual sport	213 (49.9%)	169 (47.5%)	44 (62.0%)	
Team sport	214 (50.1%)	187 (52.5%)	27 (38.0%)	
**Duration of impairment**				
Not a para-athlete		356 (100.0%)		
0–5 Years			1 (1.4%)	
5–10 Years			14 (19.7%)	
10–15 Years			13 (18.3%)	
15 + Years			10 (14.1%)	
Since birth			33 (46.5%)	

**TABLE 2:** Mental health symptoms among currently competing elite non-para athletes and para athletes.

	Total (*N* = 427)	Non-para athletes (*N* = 356)	Para athletes (*N* = 71)	*P-value*
**Mental health symptoms (GHQ-28)**				0.613
Mean (*SD*)	22.6 (12.8)	22.5 (12.6)	23.3 (14.0)	
Median (Q1, Q3)	20.0 (13.0, 29.0)	20.0 (13.0, 29.0)	19.0 (12.0, 30.0)	
Missing	44	38	6	
**General psychological distress (K-10)**				0.296
Mean (*SD*)	17.7 (7.5)	17.6 (7.3)	18.6 (8.4)	
Median (Q1, Q3)	16.0 (12.0, 20.5)	16.0 (12.0, 20.0)	17.0 (12.0, 22.0)	
Missing	48	42	6	
**Risky alcohol consumption (AUDIT-C)**				0.223
Mean (*SD*)	2.5 (2.3)	2.6 (2.3)	2.2 (2.2)	
Median (Q1, Q3)	2.0 (0.0, 4.0)	2.5 (0.0, 4.0)	2.0 (0.0, 4.0)	
Missing	54	48	6	
**Eating disorder risk (BEDA-Q)**				0.764
Mean (*SD*)	4.2 (4.0)	4.3 (3.9)	4.1 (4.2)	
Median (Q1, Q3)	3.0 (1.0, 6.0)	3.0 (1.0, 6.0)	3.0 (1.0, 5.0)	
Missing	59	52	7	

#### Para athlete specific characteristics

Of the 71 para athletes, the majority described their impairment as being physical in nature (89%), followed by visual impairment (10%), other impairments (4%) and intellectual impairment (3%). The majority of para athletes reported having had their impairment since birth (46.5%) or ≥ 10 years (32.4%). All but two para athletes were classified (97%), with one athlete yet to be classified and another awaiting reclassification. Fifty five percent of para athletes reported that issues other than classification were impacting their wellbeing, with the most common issues being equipment (28.2%), cost of travel (28.2%), venue access (28.2%) or other issues (19.7%).

### Regression analyses

Models reported here were adjusted for age and gender. A summary of risk and protective factors at the various levels of the sports ecosystem are provided in Supplementary Table 1.

#### Mental health symptoms (GHQ-28)

**FIGURE 1:**
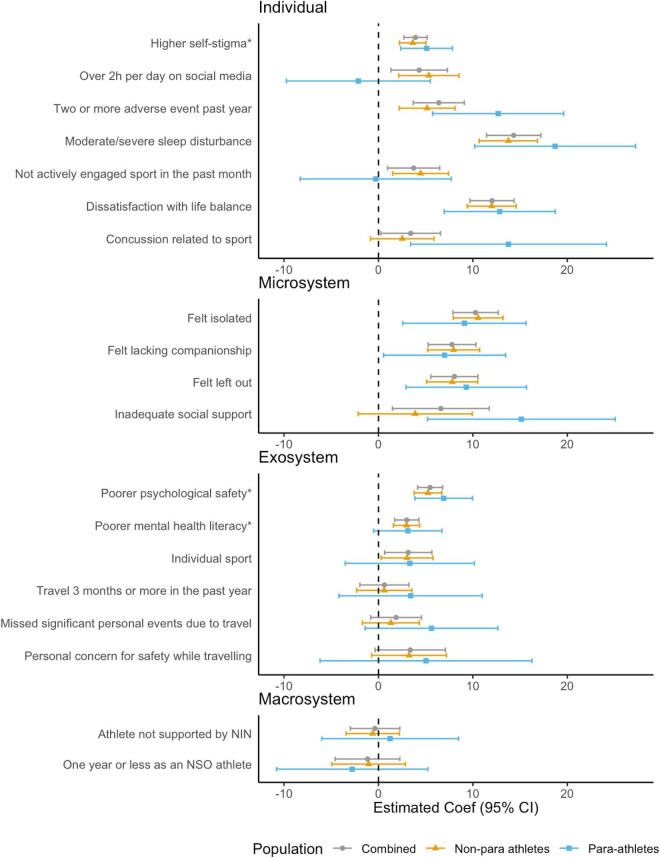
Associations between risk factors and mental health symptoms (GHQ-28) for combined, non-para and para athletes, adjusted for age and gender. *Effects associated with one standard deviation (*SD*) change in Psychological Safety subscales (Low Self-Stigma, Mentally Healthy Environment, and Mental Health Literacy).

In our model analyzing para and non-para athletes together, seven individual-, four microsystem, and four exosystem level risk factors were found to be associated with greater mental health symptoms (see Figure 1 and Supplementary Table 2). At the *individual* level, high self-stigma, moderate-to-severe sleep disturbance, experiencing two or more adverse events in the past year, being dissatisfied with life balance, and experiencing any sports-related concussion was associated with greater mental health symptoms. At the *microsystem* level, feeling isolated, lacking companionship, feeling left out, and having inadequate social support were associated with greater mental health symptoms. At the *exosystem* level, participating in a sport perceived to have low psychological safety (mentally healthy environment subscale of the SPSI), poorer mental health literacy and in an individual sport was associated with greater mental health symptoms. Mental health symptoms tended to be comparable between participants with different macro system level risk factors when controlling for age and gender.

In stratified models, looking at para and non-para athletes separately, the profile of individual risk factors among para athletes had some notable differences. For example, social media use and not being actively engaged in sport were found to be associated with mental health symptoms among non-para athletes but not para athletes. On the other hand, among para athletes, larger effects were apparent for the individual level risk factors of experiencing two or more adverse events in the past year and any sports-related concussion, as well as at the microsystem level for having inadequate social support.

When investigating whether para-athlete status had a modifying effect on the association between risk factors and mental health symptoms, there was weak evidence for an interaction effect at the individual- (social media use > 2 h, Coef = –0.43, 95% CI = –14.71, –0.15, *p* = 0.046; two or more adverse events in past year, Coef = 7.39, 0.21, 95% CI = –14.57, *p* = 0.045; concussion, 10.94, 95% CI = 0.31, 21.58, *p* = 0.044) and microsystem level (inadequate social support; Coef = 12.65, 95% CI = 1.30, 23.99, *p* = 0.030) on overall mental health symptoms, whereby being a para-athlete conferred a greater risk. This was with the exception of social media use, whereby participating in social media for > 2 h per day was associated with greater mental health symptoms for non-para athletes but not para athletes (see Supplementary Table 6).

#### General psychological distress (K-10)

**FIGURE 2:**
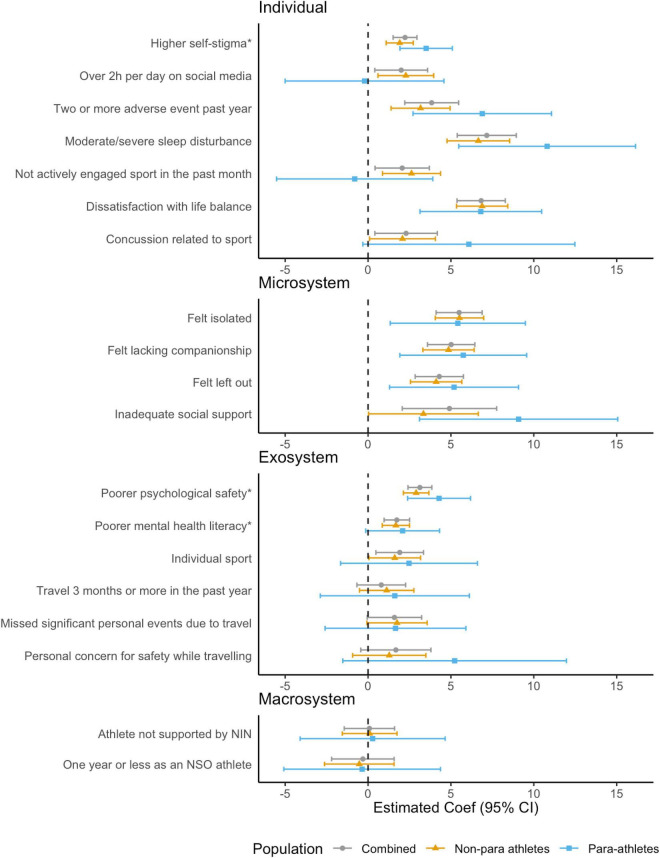
Associations between risk factors and general psychological distress (K-10) for combined, non-para and para athletes, adjusted for age and gender. *Effects associated with one standard deviation (*SD*) change in Psychological Safety subscales (Low Self-Stigma, Mentally Healthy Environment, and Mental Health Literacy).

Similar to findings for mental health symptoms, in the combined model, seven individual-, four microsystem-, and four exosystem level risk factors were found to be associated with general psychological distress (see Figure 2 and Supplementary Table 3). At *the individual* level, having high self-stigma, moderate-to-severe sleep disturbance, spending > 2 h per day on social media, experiencing two or more adverse events in the past year, not being actively engaged with their sport, being dissatisfied with their life balance and experiencing any sports-related concussion was associated with greater general psychological distress. At the *microsystem* level, feeling isolated, lacking companionship, feeling left out, and having inadequate social support were associated with greater general psychological distress. At the *exosystem* level, missing significant personal events due to travel with sport, participating in a sport perceived to have low psychological safety, having poorer mental health literacy and in an individual sport was associated with greater general psychological distress. No macro system level factors were found to be associated with general psychological distress.

In stratified models, similar to our model for mental health symptoms, the profile of individual risk factors among para athletes had some notable differences. Social media use > 2 h per day, not being actively engaged in their sport, and experiencing any sports-related concussion at the individual levels; as well as having poorer mental health literacy and missing significant personal events due to travel with sport at the exosystem level were found to be associated with general psychological distress among non-para athletes only. On the other hand, larger effects were apparent for the individual level risk factors of experiencing two or more adverse events in the past year and having inadequate social support at the microsystem level among para athletes. When investigating whether para-athlete status had a modifying effect on the association between risk factors and general psychological distress, there was weak evidence that higher self-stigma (Coef = 1.66, 95% CI = –0.07, 3.39, *p* = 0.062), experiencing two or more adverse events in the past year (Coef = 3.94, 95% CI = –0.28, 8.16, *p* = 0.069) and inadequate social support (Coef = 5.92, 95% CI = –0.34, 12.19, *p* = 0.065) were more impactful among para athletes compared with non-para athletes.

#### Risky alcohol consumption (AUDIT-C)

**FIGURE 3:**
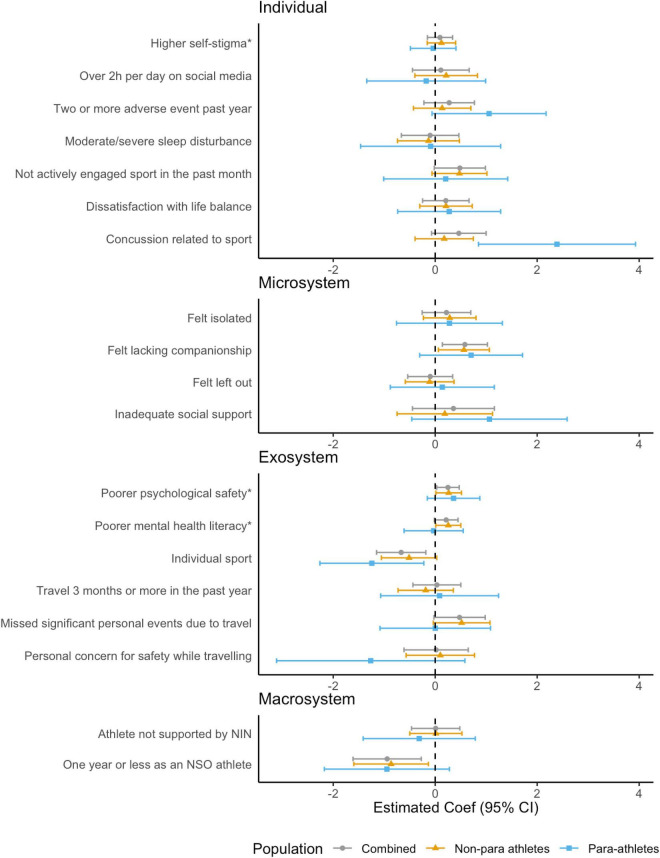
Associations between risk factors and risky alcohol consumption (AUDIT-C) for combined, non-para and para athletes, adjusted for age and gender. *Effects associated with one standard deviation (*SD*) change in Psychological Safety subscales (Low Self-Stigma, Mentally Healthy Environment, and Mental Health Literacy).

In the combined model, no individual-, one microsystem-, and three exosystem level risk factors were significantly associated with risky alcohol consumption (see Figure 3 and Supplementary Table 4). At the *microsystem* level, lacking companionship was associated with riskier alcohol consumption. At the *exosystem* level, participating in a team sport, missing significant personal events due to sport and sports perceived to have low psychological safety were associated with risky alcohol consumption. No macro system level risk factors were significantly associated with risky alcohol consumption.

In stratified models, among para athletes, experiencing any sports-related concussion was also a significant predictor of risky alcohol consumption at the individual level, however, the only other risk factor was participating in a team sport. When investigating whether para-athlete status had a modifying effect on the association between risk factors and risky alcohol consumption, a significant interaction effect was found for experiencing any sports related concussion (Coef = 2.22, 95% CI = 0.43, 4.02, *p* = 0.016), whereby being a para-athlete conferred a greater risk.

#### Eating disorder risk (BEDA-Q)

**FIGURE 4:**
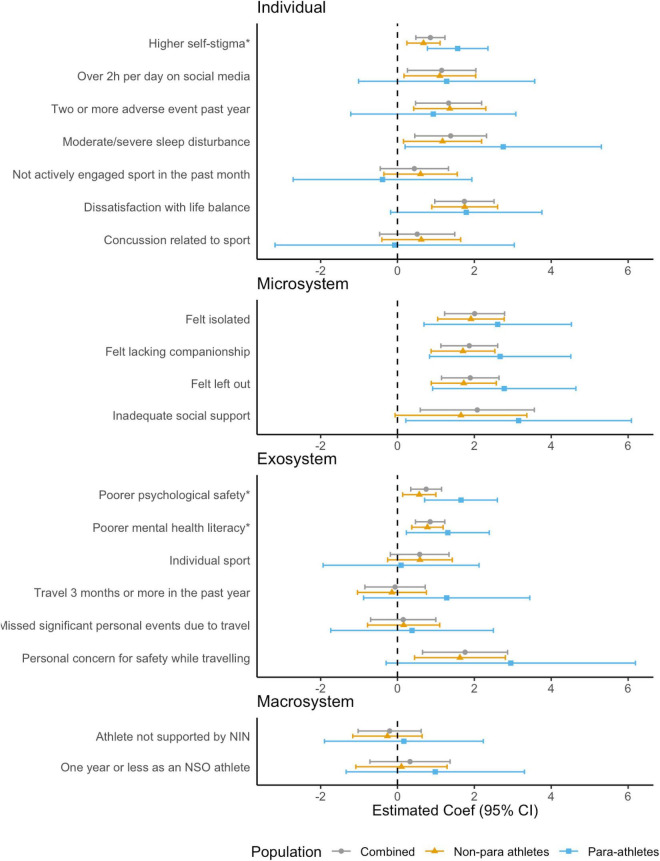
Associations between risk factors and eating disorder risk (BEDA-Q) for combined, non-para and para athletes, adjusted for age and gender. *Effects associated with one standard deviation (*SD*) change in Psychological Safety subscales (Low Self-Stigma, Mentally Healthy Environment, and Mental Health Literacy).

In the combined model, five individual-, four microsystem-, and three exosystem level risk factors were significantly associated with eating disorder risk (see Figure 4 and Supplementary Table 5). At the *individual* level, having high self-stigma, moderate-to-severe sleep disturbance, spending > 2 h per day on social media, experiencing two or more adverse events in the past year, and being dissatisfied with their life balance were significantly associated with eating disorder risk. At the *microsystem* level, feeling isolated, lacking companionship, feeling left out, and having inadequate social support were associated with greater eating disorder risk. At the *exosystem* level, participating in a sport perceived to have low psychological safety, having poorer mental health literacy and having personal concern for safety while traveling for sport were associated with eating disorder risk. No macro system level risk factors were found to be associated with eating disorder risk.

In stratified models, among para athletes, the profile of risk factors was more apparent at the microsystem level, where all four risk factors were estimated to have a higher level of association (larger point estimate, although wider confidence interval due to smaller sample size) with eating disorder risk compared with non-para athletes. While at the individual level, only self-stigma (strong evidence) and moderate/severe sleep disturbance (weak evidence) were associated with eating disorder risk. When investigating whether para-athlete status had a modifying effect on the association between risk factors and eating disorder risk, there was weak evidence supporting interaction effects for higher self-stigma (Coef = 0.92, 95% CI = 0.00, 1.84, *p* = 0.051) and poor psychological safety (Coef = 1.14, 95% CI = 0.11, 2.17, *p* = 0.31), whereby being a para-athlete conferred a greater risk.

## Discussion

This study applied a socioecological approach to identify risk and protective factors for a range of mental health outcomes across multiple levels (individual, microsystem, exosystem, and macrosystem levels) of the elite sport “ecosystem” for both para and non-para athletes. While no significant differences in the rates of mental health problems were observed between para and non-para athletes, both differences and similarities in risk and protective factor profiles were found across the multiple levels of the sports-ecosystem. Weak evidence was also found to support the hypothesis that certain risk factors, including experiencing two or more adverse events in the past year, sports related concussion, high self-stigma, inadequate social support and low psychological safety conferred a greater risk for poorer mental health outcomes for para athletes in particular. Our findings contribute to the scant literature investigating correlates of para athlete mental health and point to the need for targeted prevention and intervention strategies tailored to athlete subpopulations, such as para athletes. Our findings also provide new evidence on the role of risk and protective factors occurring at broader levels of the sports ecosystem, including those related to sporting organization culture (e.g., psychological safety).

A range of risk and protective factors were found to be associated with the four mental health outcomes assessed in the current study for both para and non-para athletes. Perhaps not surprisingly, similar profiles of risk factors across the various levels of the sports ecosystem were found for mental health symptoms and general psychological distress, where risk factors tended to cluster around individual and microsystem level factors. The most consistent correlates at the microsystem level being those relating to social isolation (e.g., feeling left out, feeling isolated, lacking companionship), and at the individual level, reporting higher self-stigma, experiencing moderate-to-severe sleep disturbance, two or more adverse events in the past year and dissatisfaction with life balance. These finding were somewhat consistent with prior research into athlete populations involving both para and non-para athletes (Swartz et al., 2019; Kuettel and Larsen, 2020; Purcell et al., 2020; Walton et al., 2021). At the exosystem level, participating in a sport perceived to have greater psychological safety was a consistent protective factor for both mental health symptoms and general psychological distress, but also for risky alcohol consumption (with the exception of para athletes) and eating disorder risk.

While there has been growing interest in the application of psychological safety in the elite sport setting, to date, little published evidence exists to inform how psychologically safe environments may influence mental health outcomes among those operating in such environments. However, evidence drawn from other high performance environments, including corporate and medical sectors, have shown psychologically safe environments to be associated with improved performance at both the individual and team level, as well as with work engagement, commitment, satisfaction and teamwork (Frazier et al., 2017); all factors that may positively influence mental health. Emerging evidence drawn from the sports literature supports these earlier findings, where it was shown that sporting environments that were perceived to be psychological safe encouraged teamwork and satisfaction with team performance, and acted as a buffer against athlete burnout (Fransen et al., 2020). Similarly, in their recent scoping review investigating risk and protective factors for mental health in elite athletes, Kuettel and Larsen (2020) found that a trusting and mastery-orientated climate (Lundqvist and Raglin, 2015), including settings characteristic of confidentiality and trust in coach (Gulliver et al., 2012; Lundqvist and Sandin, 2014), and encouragement of others toward help-seeking (Gulliver et al., 2012) were found to be positively related to athlete mental health. Work by our group has further demonstrated that the sports psychological safety domains of the SPSI were inversely related to general and athlete-specific psychological distress, and positively associated with psychological wellbeing among elite athletes (Rice et al., 2022). The current study extends these initial findings by further demonstrating a relationship between poorer psychological safety and greater mental health symptoms, risky alcohol consumption and eating disorder risk for both para and non-para athletes.

Developing and maintaining sporting environments that enhance psychological safety is likely to be associated with indicators of better mental health among those operating in elite sporting organizations. Our findings indicate that psychological safety may act as a protective factor against mental ill-health, operating at the broader organizational (exosystem) level. Cultures promoting psychological safety are characterized by having a sense of confidence in taking interpersonal risks or making mistakes without fear of negative consequences (Edmondson, 1999), and this may include perceived organizational support for disclosure of mental health problems and proactive support for their management (Rice et al., 2022). Yet, common features of elite sporting environments are often at odds with such cultures. For example, those operating in elite sporting environments often prize mental toughness (with a narrow view of this concept, often characteristic of emotion suppression and poor self-awareness), “win at all costs” attitudes, put reputational needs of the sport over the health and safety of individuals, and can encourage cultures where open disclosure of vulnerabilities, including mental health symptoms, is implicitly or explicitly stigmatized, discouraged or inhibited (Coulter et al., 2016). The latter is often cited as being due to fear of reputational damage to the sport or career-related repercussions, including loss of selection, opportunities to compete or contract renewal for individual athletes (Watson and White, 2007). However, we argue that upholding the trust of a sporting organization’s brand can be consistent with caring for athlete mental health (and the mental health of all stakeholder’s operating in these environments) and providing a safe environment. Psychological safety is positively influenced by organizational policies and procedures (Zadow et al., 2019), and there is an opportunity in elite sport to target these broader organizational level correlates and antecedents of mental health outcomes. This includes ensuring leaders in sporting organizations (both executive and sporting staff) are informed and supported to foster psychologically safe environments, which may in turn facilitate better mental health.

While no differences were found between para and non-para athletes across any of the mental ill-health outcomes, the rates of probable caseness reported in the current study are higher than that found in similar previous cohorts (Purcell et al., 2020; Olive et al., 2021). This increase may relate to the timing of the survey, which commenced during the early stages of COVID-19. Perhaps more pertinent to the current cohort was that the survey was also administered during the time that postponement of the 2020 Tokyo Olympic and Paralympic Games was announced. It is therefore possible that this announcement (and the uncertainty leading up to the announcement) had a negative impact on the mental health of athletes striving to compete at these events. While many of the same risk factors investigated in the current study were found to be associated with indicators of poorer mental health for both para and non-para athletes, we also found weak evidence of a modifying effect of athlete subtype across each of the mental health outcomes, whereby being a para athletes conferred a greater risk for poorer mental health outcomes. It may be argued that current mental health programs in elite sport have largely been developed based on evidence relating to non-para athletes, with these same programs then offered to para athlete cohorts, without much adaptation, based on an assumption that para athletes have the same needs as non-para-athletes. The current research suggests that this assumption may not be entirely accurate and that para athletes may benefit from programs that are designed with their specific needs in mind. For example, para athletes may need different protocols following experiences of concussion, not just in terms of return to play protocols (Weiler et al., 2021) but also in considering how their mental health is supported in the context of other medical complexities and life stressors. Similarly, and notwithstanding the large degree of heterogeneity among para athletes, both prevention and early intervention programs developed with para athletes in mind would do well to consider how the experience of ongoing daily stressors relating to an impairment (e.g., issue of accessibility when attending medical/sports-related appointments) and the required internal resources needed to manage such stressors, may impact on para athlete mental health. In line with coping theory (Lazarus and Folkman, 1984), this type of chronic stress may mean that less emotional/internal resources are available when/if an adverse event does occur, which may leaving para athletes more vulnerable. Programs that support the development of internal personal coping resources and external social support (in line with stress buffering theory; Alloway and Bebbington, 1987), may be well suited for this subpopulation. However, more research is needed to determine the best approach.

In addressing why para athlete status was an effect modifier of these relationships, while speculative (but in a similar vein to our suggestions on prevention/early intervention program development), we propose that this may be due to differences in the social context or lived experience of individuals living with an impairment compared to non-para athletes, or differences in the systems supporting them. For example, it has been suggested that para athletes are likely to experience a range of sport-specific and impairment-specific stressors that do not commonly affect elite athletes without disabilities, and this has the potential to compromise their personal wellbeing (Campbell and Jones, 2002; Macdougall et al., 2015). In the current cohort, para athletes were significantly more likely to report having experienced discrimination, both in the year prior to the survey (14% para vs. 3% non-para athletes) and across their lifetime (48% para vs. 15% non-para athletes). While trauma is an important concern for all athletes, it has been suggested that a substantial proportion of Paralympic athletes have acquired disabilities directly resulting from trauma (International Paralympic Committee, 2019). Given the evidence on the negative effect of cumulative adversity on mental ill-health (Turner and Lloyd, 1995; Hughes et al., 2017), it may be the case that experiencing subsequent adverse events may disproportionately affect mental health outcomes among para athletes due to an increased likelihood of earlier traumatic experiences and the negative cumulative effect of subsequent adverse events. While trauma was not assessed in the current study, we did assess a range of adverse experiences across the lifetime and found that para athletes reported a significantly greater number of adverse events experienced across their lifetime compared to non-para athletes (*M* para = 4.4, *SD* = 3.0 vs. non-para athletes *M* = 3.6, *SD* = 2.5), but there was no evidence of a significant difference in the frequency at which athletes endorsed having ever experienced an injury/illness in their lifetime (35% para vs. 29% non-para athletes). It is also worth noting that in the current para athlete cohort, almost half reported having had their impairment since birth (46.5%), so the cumulative adversity hypothesis suggested here may not be the experience of all para athletes. There is a need for further research to understand how trauma may affect para athletes, and the extent to which symptoms related to trauma exposure(s) are prevalent in this population.

Similarly, with regards to sports related concussion conferring a greater risk to some areas of mental ill-health among para athletes, this may relate to the notion that para athletes in general are medically more complex than non-para-athletes and these complexities may be contributing to the modifying effect observed in the current study. At the very least, and in line with the recent position statement on concussion in para sport released by the Concussion in Para Sport Group (Weiler et al., 2021), these findings point to the urgent need for more para-specific concussion research to inform policy on prevention and early intervention efforts when considering risk any conferred risk to mental health among this subpopulation. Also worthy of further investigation is the comparison between rates of mental health among para athletes and individuals with an impairment from the general population. Alternatively, being involved in high performance sport may serve as a protective factor from mental ill-health for para athletes compared to individuals with impairments from the general population, where rates of mental health have been observed to be much higher (Watson et al., 2014). However, these data among the general population are largely based on individuals with intellectual impairments and more research is required to determine any differences between para athletes and individuals from the general population with other types of impairments. Nonetheless, the findings reported here of a modifying effect of athletes subtype, which indicates a subset.

## Strengths and limitations

Strengths of this study included the assessment of a greater range of risk factors across varying levels of the sports ecosystem, as well as multiple mental health outcomes, and the adjustment of potentially confounding variables. Limitations include that the survey design of the study may introduce participation bias. Despite this limitation, the sample was broadly representative of the eligible population in relation to para-status and mean age, except for over representation of women athletes, which were controlled in regression models. Careful efforts were made during the survey design to maximize the reliability of the data, with the anonymous nature of the survey likely to have facilitated this by limiting social desirability bias. The sample size of para athletes is small relative to non-para athletes, which may limit any conclusions regarding the relationship between key correlates and outcomes. A few coefficients (sleep disturbance, concussion related to sport, and any personal concern for safety while traveling) estimated in the stratified regression models for para athletes need to be interpreted with care due to small numbers. Future studies would benefit from the inclusion of a broader range of risk factors relating to environmental, organizational and broader sporting contextual factors. Finally, the cross-sectional nature of the study limits the ability to capture longitudinal or causal associations. Similarly, data was not directly collected on mental health history/prior diagnosis.

## Conclusion

Risk factors occurring across various levels of the sports ecosystem, including individual, interpersonal and organizational level risk factors were found to be associated with a range of poorer mental health outcomes, including greater mental health symptoms, general psychological distress, risky alcohol consumption and eating disorder risk. While no significant differences were observed between the rates of mental health problems between para- and non-para athletes, weak evidence supports the premise that certain risk factors, including experiencing two or more adverse events in the past year, sports related concussion, high self-stigma, inadequate social support and low psychological safety conferred a greater risk for poorer mental health outcomes for para athletes in particular. Our findings contribute to the scant literature investigating correlates of para athlete mental health and provides new evidence on the role of risk factors occurring at broader levels of the sports eco-system, including those related to sporting organization culture (e.g., psychological safety).

## Data availability statement

The datasets presented in this article are not readily available because the ethics committee has not approved the sharing of data in this manner. Requests to access the datasets should be directed to the corresponding author.

## Ethics statement

The studies involving human participants were reviewed and approved by The University of Melbourne Human Ethics Research Committee. Written informed consent from the participants or their legal guardian/next of kin was not required to participate in this study in accordance with the national legislation and the institutional requirements.

## Author contributions

LO, RP, and SR conceived the idea for the current study. RP, SR, MB, MC, and GC contributed to the survey design. RP and SR were responsible for the implementation of the survey. CG and LO conducted the analyses. LO drafted the initial manuscript. All authors contributed to the data interpretation and revision of the final manuscript.
