# Supplementary material for: Risk and protective factors for mental ill-health in elite para- and non-para athletes
Source: Front Psychol. 2022 Sep 2;13:939087. doi: 10.3389/fpsyg.2022.939087 (PMC9480503; doi:10.3389/fpsyg.2022.939087)
Supplement: Supplementary file 1 [file Data_Sheet_1.docx]

**Supplemental Tables**

**Table S1**

Descriptive information for risk and protective factors for non-para and para athletes

|  | **Total (N=427)** | **Non-para athletes (N=356)** | **Para athletes (N=71)** | **p value** |
| --- | --- | --- | --- | --- |
| **Individual level factors** | |  |  |  |
| **Psychological Safety - Low Self-Stigma subscale** |  |  |  | 0.684 |
| Mean (SD) | 7.4 (2.7) | 7.4 (2.6) | 7.5 (3.0) |  |
| Median (Q1, Q3) | 8 (6, 9) | 8 (6, 9) | 7.5 (6, 10) |  |
| Missing | 85 | 74 | 11 |  |
| **Hours per day spent on social media** |  |  |  | 0.565 |
| 0-2 hrs | 239 (64.6%) | 195 (63.9%) | 44 (67.7%) |  |
| >2 hrs | 131 (35.4%) | 110 (36.1%) | 21 (32.3%) |  |
| Missing | 57 | 51 | 6 |  |
| **Number of adverse events (past year)** |  |  |  | 0.909 |
| 0-1 | 290 (70.7%) | 243 (70.8%) | 47 (70.1%) |  |
| 2+ | 120 (29.3%) | 100 (29.2%) | 20 (29.9%) |  |
| Missing | 17 | 13 | 4 |  |
| **Sleep disturbance** |  |  |  | 0.344 |
| None/below mild/mild sleep disturbance | 299 (80.4%) | 244 (79.5%) | 55 (84.6%) |  |
| Moderate/severe sleep disturbance | 73 (19.6%) | 63 (20.5%) | 10 (15.4%) |  |
| Missing | 55 | 49 | 6 |  |
| **Main activity related to sport (past month)** |  |  |  | 0.172 |
| Actively engaged sport | 302 (70.7%) | 247 (69.4%) | 55 (77.5%) |  |
| Not actively engaged sport | 125 (29.3%) | 109 (30.6%) | 16 (22.5%) |  |
| **Satisfaction with life balance** |  |  |  | 0.363 |
| Yes | 239 (61.9%) | 202 (62.9%) | 37 (56.9%) |  |
| No | 147 (38.1%) | 119 (37.1%) | 28 (43.1%) |  |
| Missing | 41 | 35 | 6 |  |
| **Any concussion related to sport** |  |  |  | 0.016 |
| No | 340 (79.6%) | 276 (77.5%) | 64 (90.1%) |  |
| Yes | 87 (20.4%) | 80 (22.5%) | 7 (9.9%) |  |
| **Microsystem factors** |  |  |  |  |
| **Frequency feeling isolated** |  |  |  | 0.162 |
| Hardly ever | 159 (43.8%) | 136 (45.5%) | 23 (35.9%) |  |
| Some of the time/often | 204 (56.2%) | 163 (54.5%) | 41 (64.1%) |  |
| Missing | 64 | 57 | 7 |  |
| **Frequency feeling of lacking companionship** |  |  |  | 0.898 |
| Hardly ever | 161 (44.5%) | 133 (44.6%) | 28 (43.8%) |  |
| Some of the time/often | 201 (55.5%) | 165 (55.4%) | 36 (56.2%) |  |
| Missing | 65 | 58 | 7 |  |
| **Frequency feeling left out** |  |  |  | 0.508 |
| Hardly ever | 161 (44.4%) | 135 (45.2%) | 26 (40.6%) |  |
| Some of the time/often | 202 (55.6%) | 164 (54.8%) | 38 (59.4%) |  |
| Missing | 64 | 57 | 7 |  |
| **Adequate social support** |  |  |  | 0.132 |
| Yes | 327 (91.9%) | 273 (92.9%) | 54 (87.1%) |  |
| No | 29 (8.1%) | 21 (7.1%) | 8 (12.9%) |  |
| Missing | 71 | 62 | 9 |  |
| **Exosystem factors** |  |  |  |  |
| **Psychological Safety - Mentally Healthy Environment subscale** | |  |  | 0.028 |
| Mean (SD) | 10.2 (3.6) | 10.4 (3.6) | 9.3 (3.6) |  |
| Median (Q1, Q3) | 11 (8, 12) | 11 (9 ,12) | 9.5 (8, 12) |  |
| Missing | 84 | 75 | 9 |  |
| **Psychological Safety - Mental Health Literacy subscale** | |  |  | 0.864 |
| Mean (SD) | 10.9 (2.9) | 10.9 (2.9) | 10.9 (2.5) |  |
| Median (Q1, Q3) | 11 (9.5, 12) | 11 (9, 12) | 11(10, 12) |  |
| Missing | 84 | 74 | 10 |  |
| **Sport Type** |  |  |  | 0.026 |
| Team sport | 214 (50.1%) | 187 (52.5%) | 27 (38.0%) |  |
| Individual sport | 213 (49.9%) | 169 (47.5%) | 44 (62.0%) |  |
| **Time travelling due to sport** |  |  |  | < 0.001 |
| 2 months or less | 231 (54.1%) | 178 (50.0%) | 53 (74.6%) |  |
| 3 months+ | 196 (45.9%) | 178 (50.0%) | 18 (25.4%) |  |
| **Missed significant personal events due to travel** | |  |  | 0.988 |
| No | 138 (32.3%) | 115 (32.3%) | 23 (32.4%) |  |
| Yes | 289 (67.7%) | 241 (67.7%) | 48 (67.6%) |  |
| **Any personal concern for safety while travelling** | |  |  | 0.316 |
| No | 369 (86.4%) | 305 (85.7%) | 64 (90.1%) |  |
| Yes | 58 (13.6%) | 51 (14.3%) | 7 (9.9%) |  |
| **Macrosystem factors** |  |  |  |  |
| **Athlete supported by NIN** |  |  |  | 0.020 |
| Yes | 254 (59.5%) | 203 (57.0%) | 51 (71.8%) |  |
| No | 173 (40.5%) | 153 (43.0%) | 20 (28.2%) |  |
| **Number of years as an NSO athlete** |  |  |  | 0.971 |
| 1 year or less | 88 (25.2%) | 72 (25.2%) | 16 (25.4%) |  |
| 2 year + | 261 (74.8%) | 214 (74.8%) | 47 (74.6%) |  |
| Missing | 78 | 70 | 8 |  |

**Table S2**

Associations between risk factors and mental health symptoms (GHQ-28) for combined, non-para and para athletes, adjusted for age and gender

|  | Combined | | Non-para athletes | | Para athletes | |
| --- | --- | --- | --- | --- | --- | --- |
| **Risk factors** | **Coef (95% CI)** | **p-value** | **Coef (95% CI)** | **p-value** | **Coef (95% CI)** | **p-value** |
| **Individual level factors** |  |  |  |  |  |  |
| Higher self-stigma* | 3.80 (2.59 , 5.00) | <0.001 | 3.41 (2.08 , 4.75) | <0.001 | 5.35 (2.59 , 8.10) | <0.001 |
| Over 2h per day on social media | 4.29 ( 1.40 , 7.19) | 0.004 | 5.38 ( 2.29 , 8.48) | 0.001 | -2.64 (-10.62 , 5.34) | 0.519 |
| Two or more adverse event past year | 6.31 (3.51 , 9.10) | <0.001 | 5.05 (1.96 , 8.13) | 0.002 | 12.63 (5.73 , 19.52) | 0.001 |
| Moderate/severe sleep disturbance | 14.44 (11.34 , 17.55) | <0.001 | 13.76 (10.43 , 17.10) | <0.001 | 19.23 ( 9.89 , 28.56) | <0.001 |
| Not actively engaged sport in the past month | 3.63 ( 0.85 , 6.40) | 0.011 | 4.44 ( 1.43 , 7.44) | 0.004 | -0.87 (-8.92 , 7.19) | 0.834 |
| Dissatisfaction with life balance | 12.03 (9.65 , 14.41) | <0.001 | 11.95 (9.41 , 14.48) | <0.001 | 13.20 (7.04 , 19.35) | <0.001 |
| Concussion related to sport | 3.83 ( 0.60 , 7.06) | 0.021 | 2.98 (-0.44 , 6.39) | 0.089 | 13.84 ( 3.21 , 24.47) | 0.013 |
| **Microsystem factors** |  |  |  |  |  |  |
| Felt isolated | 10.18 (7.86 , 12.50) | <0.001 | 10.39 (7.90 , 12.88) | <0.001 | 9.39 (2.45 , 16.32) | 0.010 |
| Felt lacking companionship | 7.70 (5.30 , 10.10) | <0.001 | 7.81 (5.17 , 10.44) | <0.001 | 7.17 (0.53 , 13.82) | 0.039 |
| Felt left out | 7.84 (5.43 , 10.26) | <0.001 | 7.50 (4.91 , 10.09) | <0.001 | 9.67 (3.15 , 16.20) | 0.005 |
| Inadequate social support | 6.93 ( 2.22 , 11.63) | 0.004 | 3.64 (-1.92 , 9.20) | 0.201 | 16.31 ( 6.04 , 26.59) | 0.003 |
| **Exosystem factors** |  |  |  |  |  |  |
| Poorer psychological safety* | 5.18 (3.91 , 6.45) | <0.001 | 4.89 (3.54 , 6.25) | <0.001 | 6.92 (3.63 , 10.20) | <0.001 |
| Poorer mental health literacy* | 3.02 ( 1.62 , 4.42) | <0.001 | 2.98 ( 1.49 , 4.48) | <0.001 | 3.24 (-0.52 , 6.99) | 0.096 |
| Individual sport | 3.24 ( 0.72 , 5.76) | 0.012 | 3.05 ( 0.33 , 5.78) | 0.029 | 3.79 (-3.32 , 10.90) | 0.301 |
| Travel 3 months or more in the past year | 0.51 (-2.09 , 3.10) | 0.702 | 0.55 (-2.32 , 3.42) | 0.708 | 2.86 (-4.84 , 10.56) | 0.469 |
| Missed significant personal events due to travel | 1.67 (-1.22 , 4.56) | 0.259 | 1.19 (-2.05 , 4.43) | 0.473 | 5.06 (-1.99 , 12.12) | 0.164 |
| Personal concern for safety while travelling | 3.55 (-0.19 , 7.29) | 0.064 | 3.51 (-0.44 , 7.46) | 0.083 | 4.53 (-6.94 , 16.00) | 0.442 |
| **Macrosystem factors** |  |  |  |  |  |  |
| Athlete not supported by NIN | -0.35 (-2.90 , 2.21) | 0.791 | -0.62 (-3.42 , 2.18) | 0.664 | 1.37 (-6.09 , 8.84) | 0.720 |
| One year or less as an NSO athlete | -1.03 ( -4.31 , 2.25) | 0.539 | -0.95 ( -4.59 , 2.68) | 0.608 | -2.48 (-10.86 , 5.89) | 0.565 |

***** Effects associated with one standard deviation (SD) change in Psychological Safety subscales (Low Self-Stigma, Mentally Healthy Environment, and Mental Health Literacy)

**Table S3**

Associations between risk factors and general psychological distress (K-10) for combined, non-para and para athletes, adjusted for age and gender

|  | Combined | | Non-para athletes | | Para athletes | |
| --- | --- | --- | --- | --- | --- | --- |
| **Risk factors** | **Coef (95% CI)** | **p-value** | **Coef (95% CI)** | **p-value** | **Coef (95% CI)** | **p-value** |
| **Individual level factors** |  |  |  |  |  |  |
| Higher self-stigma* | 2.23 (1.51 , 2.95) | <0.001 | 1.91 (1.09 , 2.73) | <0.001 | 3.50 (1.92 , 5.08) | <0.001 |
| Over 2h per day on social media | 2.00 ( 0.41 , 3.59) | 0.015 | 2.28 ( 0.60 , 3.96) | 0.008 | -0.21 (-5.00 , 4.58) | 0.931 |
| Two or more adverse event past year | 3.84 (2.21 , 5.46) | <0.001 | 3.17 (1.39 , 4.95) | 0.001 | 6.89 (2.71 , 11.07) | 0.002 |
| Moderate/severe sleep disturbance | 7.16 (5.38 , 8.94) | <0.001 | 6.65 (4.77 , 8.54) | <0.001 | 10.80 (5.47 , 16.14) | <0.001 |
| Not actively engaged sport in the past month | 2.06 ( 0.42 , 3.70) | 0.014 | 2.62 ( 0.87 , 4.38) | 0.004 | -0.81 (-5.52 , 3.91) | 0.738 |
| Dissatisfaction with life balance | 6.82 (5.37 , 8.28) | <0.001 | 6.88 (5.34 , 8.43) | <0.001 | 6.80 (3.13 , 10.47) | 0.001 |
| Concussion related to sport | 2.29 ( 0.41 , 4.18) | 0.018 | 2.08 ( 0.09 , 4.06) | 0.042 | 6.08 (-0.32 , 12.48) | 0.067 |
| **Microsystem factors** |  |  |  |  |  |  |
| Felt isolated | 5.49 (4.11 , 6.88) | <0.001 | 5.52 (4.06 , 6.98) | <0.001 | 5.41 (1.34 , 9.49) | 0.012 |
| Felt lacking companionship | 5.01 (3.59 , 6.44) | <0.001 | 4.85 (3.31 , 6.40) | <0.001 | 5.74 (1.92 , 9.57) | 0.005 |
| Felt left out | 4.30 (2.84 , 5.76) | <0.001 | 4.11 (2.56 , 5.66) | <0.001 | 5.19 (1.30 , 9.08) | 0.011 |
| Inadequate social support | 4.91 (2.06 , 7.76) | 0.001 | 3.34 (0.03 , 6.65) | 0.050 | 9.08 (3.10 , 15.06) | 0.004 |
| **Exosystem factors** |  |  |  |  |  |  |
| Poorer psychological safety* | 3.13 (2.41 , 3.85) | <0.001 | 2.90 (2.13 , 3.67) | <0.001 | 4.28 (2.39 , 6.18) | <0.001 |
| Poorer mental health literacy* | 1.74 ( 0.97 , 2.50) | <0.001 | 1.68 ( 0.86 , 2.50) | <0.001 | 2.09 (-0.14 , 4.31) | 0.072 |
| Individual sport | 1.91 ( 0.47 , 3.35) | 0.010 | 1.61 ( 0.05 , 3.17) | 0.043 | 2.47 (-1.66 , 6.60) | 0.245 |
| Travel 3 months or more in the past year | 0.80 (-0.67 , 2.27) | 0.289 | 1.12 (-0.52 , 2.76) | 0.181 | 1.61 (-2.88 , 6.11) | 0.484 |
| Missed significant personal events due to travel | 1.59 (-0.05 , 3.23) | 0.059 | 1.75 (-0.07 , 3.57) | 0.061 | 1.66 (-2.59 , 5.90) | 0.448 |
| Personal concern for safety while travelling | 1.68 (-0.44 , 3.79) | 0.121 | 1.28 (-0.93 , 3.49) | 0.257 | 5.22 (-1.52 , 11.97) | 0.134 |
| **Macrosystem factors** |  |  |  |  |  |  |
| Athlete not supported by NIN | 0.08 (-1.44 , 1.60) | 0.918 | 0.10 (-1.55 , 1.75) | 0.909 | 0.28 (-4.10 , 4.65) | 0.902 |
| One year or less as an NSO athlete | -0.31 (-2.20 , 1.57) | 0.746 | -0.53 (-2.63 , 1.57) | 0.623 | -0.36 (-5.08 , 4.37) | 0.883 |

***** Effects associated with one standard deviation (SD) change in Psychological Safety subscales (Low Self-Stigma, Mentally Healthy Environment, and Mental Health Literacy)

**Table S4**

Associations between risk factors and risky alcohol consumption (AUDIT-C) for combined, non-para and para athletes, adjusted for age and gender

|  | Combined | | Non-para athletes | | Para athletes | |
| --- | --- | --- | --- | --- | --- | --- |
| **Risk factors** | **Coef (95% CI)** | **p-value** | **Coef (95% CI)** | **p-value** | **Coef (95% CI)** | **p-value** |
| **Individual level factors** |  |  |  |  |  |  |
| Higher self-stigma* | 0.09 (-0.15 , 0.34) | 0.460 | 0.12 (-0.16 , 0.40) | 0.393 | -0.04 (-0.49 , 0.41) | 0.863 |
| Over 2h per day on social media | 0.11 (-0.45 , 0.67) | 0.699 | 0.22 (-0.40 , 0.83) | 0.495 | -0.18 (-1.34 , 0.99) | 0.768 |
| Two or more adverse event past year | 0.27 (-0.22 , 0.77) | 0.280 | 0.14 (-0.43 , 0.70) | 0.636 | 1.06 (-0.06 , 2.18) | 0.070 |
| Moderate/severe sleep disturbance | -0.10 (-0.66 , 0.46) | 0.728 | -0.13 (-0.74 , 0.47) | 0.670 | -0.09 (-1.46 , 1.28) | 0.898 |
| Not actively engaged sport in the past month | 0.48 (-0.02 , 0.99) | 0.061 | 0.48 (-0.06 , 1.01) | 0.085 | 0.21 (-1.01 , 1.42) | 0.741 |
| Dissatisfaction with life balance | 0.21 (-0.25 , 0.66) | 0.371 | 0.21 (-0.30 , 0.73) | 0.421 | 0.27 (-0.74 , 1.28) | 0.597 |
| Concussion related to sport | 0.46 (-0.07 , 1.00) | 0.091 | 0.18 (-0.40 , 0.75) | 0.546 | 2.39 ( 0.85 , 3.93) | 0.003 |
| **Microsystem factors** |  |  |  |  |  |  |
| Felt isolated | 0.22 (-0.26 , 0.70) | 0.367 | 0.28 (-0.23 , 0.80) | 0.282 | 0.28 (-0.76 , 1.32) | 0.600 |
| Felt lacking companionship | 0.58 ( 0.14 , 1.02) | 0.010 | 0.56 ( 0.06 , 1.06) | 0.029 | 0.70 (-0.30 , 1.71) | 0.177 |
| Felt left out | -0.10 (-0.54 , 0.34) | 0.664 | -0.11 (-0.59 , 0.37) | 0.656 | 0.14 (-0.88 , 1.16) | 0.790 |
| Inadequate social support | 0.36 (-0.45 , 1.16) | 0.384 | 0.19 (-0.75 , 1.12) | 0.694 | 1.06 (-0.46 , 2.59) | 0.176 |
| **Exosystem factors** |  |  |  |  |  |  |
| Poorer psychological safety* | 0.25 ( 0.03 , 0.47) | 0.027 | 0.26 ( 0.01 , 0.52) | 0.041 | 0.36 (-0.16 , 0.88) | 0.177 |
| Poorer mental health literacy* | 0.22 (-0.02 , 0.45) | 0.071 | 0.26 ( 0.01 , 0.50) | 0.043 | -0.03 (-0.61 , 0.55) | 0.918 |
| Individual sport | -0.67 (-1.15 , -0.18) | 0.008 | -0.51 (-1.06 , 0.03) | 0.068 | -1.24 (-2.26 , -0.22) | 0.020 |
| Travel 3 months or more in the past year | 0.03 (-0.44 , 0.50) | 0.892 | -0.19 (-0.73 , 0.36) | 0.502 | 0.09 (-1.07 , 1.24) | 0.884 |
| Missed significant personal events due to travel | 0.48 (-0.03 , 0.98) | 0.065 | 0.52 (-0.04 , 1.07) | 0.070 | 0.00 (-1.08 , 1.08) | 0.998 |
| Personal concern for safety while travelling | 0.02 (-0.61 , 0.65) | 0.955 | 0.10 (-0.57 , 0.77) | 0.770 | -1.26 (-3.11 , 0.58) | 0.187 |
| **Macrosystem factors** |  |  |  |  |  |  |
| Athlete not supported by NIN | 0.01 (-0.46 , 0.48) | 0.970 | 0.01 (-0.50 , 0.52) | 0.960 | -0.31 (-1.41 , 0.79) | 0.579 |
| One year or less as an NSO athlete | -0.94 (-1.61 , -0.27) | 0.010 | -0.87 (-1.60 , -0.13) | 0.027 | -0.95 (-2.18 , 0.28) | 0.130 |

***** Effects associated with one standard deviation (SD) change in Psychological Safety subscales (Low Self-Stigma, Mentally Healthy Environment, and Mental Health Literacy).

**Table S5**

Associations between risk factors and eating disorder risk (BEDA-Q) for combined, non-para and para athletes, adjusted for age and gender

|  | Combined | | Non-para athletes | | Para athletes | |
| --- | --- | --- | --- | --- | --- | --- |
| **Risk factors** | **Coef (95% CI)** | **p-value** | **Coef (95% CI)** | **p-value** | **Coef (95% CI)** | **p-value** |
| **Individual level factors** |  |  |  |  |  |  |
| Higher self-stigma* | 0.86 (0.48 , 1.23) | <0.001 | 0.68 (0.24 , 1.11) | 0.003 | 1.57 (0.78 , 2.35) | <0.001 |
| Over 2h per day on social media | 1.15 ( 0.26 , 2.04) | 0.013 | 1.10 ( 0.17 , 2.04) | 0.022 | 1.28 (-1.01 , 3.57) | 0.279 |
| Two or more adverse event past year | 1.33 ( 0.47 , 2.19) | 0.003 | 1.36 ( 0.42 , 2.30) | 0.005 | 0.93 (-1.22 , 3.08) | 0.399 |
| Moderate/severe sleep disturbance | 1.38 (0.45 , 2.32) | 0.004 | 1.17 (0.16 , 2.19) | 0.025 | 2.75 (0.20 , 5.31) | 0.039 |
| Not actively engaged sport in the past month | 0.44 (-0.45 , 1.33) | 0.335 | 0.60 (-0.35 , 1.56) | 0.220 | -0.39 (-2.71 , 1.94) | 0.744 |
| Dissatisfaction with life balance | 1.74 ( 0.97 , 2.51) | <0.001 | 1.75 ( 0.89 , 2.61) | <0.001 | 1.79 (-0.18 , 3.76) | 0.081 |
| Concussion related to sport | 0.51 (-0.46 , 1.49) | 0.304 | 0.62 (-0.41 , 1.64) | 0.238 | -0.07 (-3.19 , 3.04) | 0.963 |
| **Microsystem factors** |  |  |  |  |  |  |
| Felt isolated | 2.01 (1.23 , 2.79) | <0.001 | 1.91 (1.05 , 2.78) | <0.001 | 2.61 (0.69 , 4.52) | 0.010 |
| Felt lacking companionship | 1.87 (1.13 , 2.61) | <0.001 | 1.70 (0.87 , 2.53) | <0.001 | 2.67 (0.83 , 4.51) | 0.006 |
| Felt left out | 1.89 (1.15 , 2.64) | <0.001 | 1.72 (0.88 , 2.57) | <0.001 | 2.78 (0.91 , 4.64) | 0.005 |
| Inadequate social support | 2.08 ( 0.59 , 3.56) | 0.007 | 1.65 (-0.06 , 3.37) | 0.061 | 3.15 ( 0.22 , 6.09) | 0.040 |
| **Exosystem factors** |  |  |  |  |  |  |
| Poorer psychological safety* | 0.75 (0.34 , 1.15) | <0.001 | 0.57 (0.14 , 1.00) | 0.011 | 1.65 (0.71 , 2.60) | 0.001 |
| Poorer mental health literacy* | 0.85 (0.47 , 1.23) | <0.001 | 0.78 (0.37 , 1.19) | <0.001 | 1.31 (0.23 , 2.39) | 0.021 |
| Individual sport | 0.58 (-0.19 , 1.34) | 0.139 | 0.58 (-0.26 , 1.43) | 0.174 | 0.09 (-1.94 , 2.12) | 0.929 |
| Travel 3 months or more in the past year | -0.06 (-0.85 , 0.72) | 0.875 | -0.14 (-1.04 , 0.75) | 0.752 | 1.28 (-0.88 , 3.44) | 0.250 |
| Missed significant personal events due to travel | 0.15 (-0.70 , 1.00) | 0.731 | 0.16 (-0.78 , 1.10) | 0.739 | 0.38 (-1.74 , 2.50) | 0.727 |
| Personal concern for safety while travelling | 1.76 ( 0.65 , 2.87) | 0.002 | 1.63 ( 0.44 , 2.81) | 0.008 | 2.95 (-0.30 , 6.19) | 0.080 |
| **Macrosystem factors** |  |  |  |  |  |  |
| Athlete not supported by NIN | -0.20 (-1.02 , 0.62) | 0.626 | -0.26 (-1.16 , 0.64) | 0.570 | 0.17 (-1.90 , 2.23) | 0.875 |
| One year or less as an NSO athlete | 0.33 (-0.72 , 1.37) | 0.542 | 0.10 (-1.08 , 1.29) | 0.866 | 0.98 (-1.34 , 3.30) | 0.411 |

***** Effects associated with one standard deviation (SD) change in Psychological Safety subscales (Low Self-Stigma, Mentally Healthy Environment, and Mental Health Literacy)

**Table S6**

Results from interactions models with weak (p-value <0.1) evidence of effect modification

|  | Para athletes | | Effect | | Interaction | |
| --- | --- | --- | --- | --- | --- | --- |
| **Risk factors** | **Coef (95% CI)** | **p-value** | **Coef (95% CI)** | **p-value** | **Coef (95% CI)** | **p-value** |
| **Mental health symptoms (GHQ-28)** |  |  |  |  |  |  |
| Over 2h per day on social media | 3.32 ( -1.13 , 7.76) | 0.145 | 5.31 ( 2.21 , 8.42) | 0.001 | -7.43 (-14.71 , -0.15) | 0.046 |
| Two or more adverse events past year | -1.16 (-5.26 , 2.95) | 0.581 | 5.06 ( 1.97 , 8.15) | 0.002 | 7.39 ( 0.21 , 14.57) | 0.045 |
| Concussion related to sport | 0.36 (-3.52 , 4.25) | 0.854 | 2.97 (-0.46 , 6.41) | 0.092 | 10.94 ( 0.31 , 21.58) | 0.044 |
| Inadequate social support | -0.41 (-4.13 , 3.32) | 0.831 | 3.66 (-1.89 , 9.20) | 0.197 | 12.65 ( 1.30 , 23.99) | 0.030 |
| **General psychological distress (K10)** |  |  |  |  |  |  |
| Higher self-stigma* | 1.46 (-0.46 , 3.39) | 0.137 | 1.91 ( 1.08 , 2.73) | <0.001 | 1.66 (-0.07 , 3.39) | 0.062 |
| Two or more adverse events past year | 0.15 (-2.23 , 2.54) | 0.899 | 3.17 ( 1.37 , 4.97) | 0.001 | 3.94 (-0.28 , 8.16) | 0.069 |
| Inadequate social support | 0.61 (-1.52 , 2.75) | 0.573 | 3.34 ( 0.01 , 6.67) | 0.051 | 5.92 (-0.34 , 12.19) | 0.065 |
| **Risky alcohol consumption (AUDIT-C)** |  |  |  |  |  |  |
| Concussion related to sport | -1.16 (-1.81 , -0.51) | 0.001 | 0.18 (-0.38 , 0.74) | 0.540 | 2.22 ( 0.43 , 4.02) | 0.016 |
| **Eating disorder risk (BEDA-Q)** |  |  |  |  |  |  |
| Higher self-stigma* | 0.45 (-0.56 , 1.46) | 0.378 | 0.67 ( 0.25 , 1.10) | 0.002 | 0.92 ( 0.00 , 1.84) | 0.051 |
| Poorer psychological safety* | -0.01 (-1.09 , 1.07) | 0.984 | 0.55 ( 0.12 , 0.98) | 0.012 | 1.14 ( 0.11 , 2.17) | 0.031 |

***** Effects associated with one standard deviation (SD) change in Psychological Safety subscales (Low Self-Stigma, Mentally Healthy Environment)
